# Association between ventilation–perfusion matching improvement during initial prone positioning and ICU mortality in patients with moderate to severe ARDS: a prospective two-center study

**DOI:** 10.1186/s13613-025-01489-1

**Published:** 2025-05-21

**Authors:** Rui Wang, Wancong Wang, Xiao Tang, Zhenyuan Qi, Ting Li, Yalan Liu, Hongju Li, Jican Yan, Hua Yang, Wenrui Lyu, Zhaohong Li, Bing Sun, Guifen Gan

**Affiliations:** 1 Department of Respiratory and Critical Care Medicine, Beijing Institute of Respiratory Medicine and Beijing Chao-Yang Hospital, Capital Medical University, No. 8 Gongren Tiyuchang Nanlu, Chaoyang District, Beijing, China; 2 Department of Critical Care Medicine, Affiliated Hospital of Qinghai University, No. 29 Tongren Road, Chengdong District, Xining, Qinghai China

**Keywords:** Prone position, Acute respiratory distress syndrome, Electrical impedance tomography, Ventilation-perfusion matching, Ventilator-induced lung injury

## Abstract

**Background:**

Prone positioning (PP) is widely used in patients with moderate to severe acute respiratory distress syndrome (ARDS) to reduce mortality by mitigating the risk of ventilation-induced lung injury (VILI) and enhancing ventilation-perfusion (V/Q) matching. However, patient responses to PP are variable, and the relationship between V/Q matching improvement during PP and clinical outcomes remains unclear. This study aimed to test the hypothesis that improvements in V/Q matching 4 h within the first PP are associated with reduced intensive care unit (ICU) mortality.

**Methods:**

In this two-center, prospective, observational study, regional ventilation and perfusion changes in patients with moderate to severe ARDS were evaluated using electrical impedance tomography (EIT) during the first PP session. Patients were categorized as responders or non-responders based on whether V/Q matching improved by ≥ 10% within 4 h of the first PP. The primary endpoint was ICU mortality, and the secondary endpoint was ventilator-free days at day 28.

**Results:**

A total of 77 patients were included in the study, with 46 (59.7%) classified as responders and 31 (40.3%) as non-responders. EIT revealed significant improvements in V/Q matching during PP, primarily through reduced dorsal shunt and ventral dead space. These improvements were partially sustained after resupination. Responders showed significantly lower ICU mortality (28.3% vs. 51.6%; *P* = 0.038) and more ventilator-free days at day 28 (16 [range, 0–21] days vs. 9 [0–15] days; *P* = 0.024) than non-responders. Multivariate analysis confirmed enhanced V/Q matching as an independent protective factor against mortality (OR, 0.790; 95% CI, 0.681–0.917; *P* = 0.002).

**Conclusions:**

Improvement in V/Q matching 4 h within the first PP is associated with lower ICU mortality in patients with moderate to severe ARDS. These findings underscore the importance of PP in ARDS management and highlight the potential of V/Q responsiveness in guiding individualized PP strategies.

**Trial registration:**

ClinicalTrials.Gov: NCT05765760. Registered 28 February 2023.

**Supplementary Information:**

The online version contains supplementary material available at 10.1186/s13613-025-01489-1.

## Background

Prone positioning (PP) is widely used in the treatment of moderate to severe acute respiratory distress syndrome (ARDS) because it improves oxygenation and reduces mortality [1–3]. It redistributes ventilation, promotes alveolar recruitment, reduces hyperinflation, and enhances lung homogeneity, lowering the risk of ventilation-induced lung injury (VILI) [4]. The recruitment of dorsal lung regions, which also receive the majority of pulmonary blood flow, improves ventilation-perfusion (V/Q) matching, thereby enhancing oxygenation [5]. Previous studies have suggested that PP can improve oxygenation in 70-80% of ARDS patients; however, this benefit is not universal [6–9]. Therefore, it can be inferred that PP may not improve V/Q matching in every patient. Over the years, the absence of simple bedside methods has hindered the collection of direct evidence from patients.

Electrical impedance tomography (EIT) is a non-invasive, radiation-free, and real-time monitoring technique that precisely assesses regional ventilation and perfusion distribution [10–12]. The evaluation of ventilation using EIT is already well-established. However, perfusion assessment is still relatively new and most commonly relies on using hypertonic saline as a “conductive” agent, which has been recently validated in experimental studies [13–15]. This approach is efficient for monitoring dynamic responses to maneuvers such as PP, enhancing the physiological understanding and management of ARDS at the bedside [12]. In theory, PP enhances V/Q matching by reducing dorsal shunt through increased ventilation in well-perfused regions while decreasing ventral dead space by lowering ventilation in poorly perfused areas [16]. Few studies have explored the impact of PP on V/Q matching in ARDS patients, with limitations such as small sample sizes, a focus on the early effects of PP, and a lack of assessment of V/Q changes after returning to the supine position [17–20]. Moreover, the relationship between changes in V/Q matching after PP and survival remains unclear.

We hypothesized that enhanced V/Q matching after the first session of PP might correlate with better outcomes in patients with moderate to severe ARDS. Therefore, we conducted a prospective observational study at two centers to investigate the relationship between V/Q matching improvement 4 h within the first PP and intensive care unit (ICU) mortality.

## Methods

### Study design and patients

This prospective observational study was conducted at two tertiary hospitals in China: Beijing Chao-Yang Hospital and Qinghai University Affiliated Hospital. The research protocol received authorization from the Institutional Review Board at each participating center and was registered on ClinicalTrials.gov (NCT05765760). This study was conducted in accordance with the ethical principles outlined in the Declaration of Helsinki (2013 revised edition). Written informed consent was obtained from the legal guardians because the patients were unable to provide consent at the time of inclusion.

We included patients who met the following criteria: (1) fulfillment of the diagnostic criteria for moderate to severe ARDS according to the Berlin definition, with a ratio of partial pressure of arterial oxygen (PaO₂) to fraction of inspired oxygen (FiO₂) < 150 mm Hg under optimal mechanical ventilation [21]; and (2) placement a central venous catheter in the jugular or subclavian vein for treatment based on a clinical decision at enrollment.

Patients were excluded if they met any of the following criteria: (1) age less than 18 years; (2) pregnancy; (3) body mass index (BMI) greater than 35 kg/m²; (4) receipt of invasive mechanical ventilation (IMV) for more than 48 h; (5) severe hemodynamic instability (e.g., mean arterial pressure < 65 mmHg and norepinephrine > 0.5 µg/kg/min); (6) immediate need for venovenous extracorporeal membrane oxygenation (VV-ECMO) therapy; or (7) contraindications to PP or EIT (e.g., facial or neck trauma, spinal instability, pacemaker, implantable defibrillator, chest and abdominal skin lesions).

### Study protocol

The following patient characteristics were collected at enrollment: age, sex, BMI, clinical scores, etiology and severity of ARDS, underlying comorbidities, duration of IMV before PP, hemodynamic status, arterial blood gas, and ventilatory variables.

All patients received volume-controlled mechanical ventilation with standardized settings: tidal volume set at 6–8 mL/kg of predicted body weight, respiratory rate adjusted to maintain a pH of 7.35 to 7.45, positive end-expiratory pressure (PEEP) determined after a recruitment maneuver and decremental titration to achieve the highest respiratory system compliance in the supine position and kept constant during PP, and FiO_2_ adjusted to maintain oxygen saturation (SpO_2_) of 93–96%. Plateau pressure and total PEEP were obtained during end-inspiratory and end-expiratory pauses at zero-flow points. Driving pressure was determined as the difference between plateau pressure and total PEEP, and respiratory system compliance was calculated as tidal volume divided by driving pressure. All patients were sedated and under neuromuscular blockade during measurements.

For the first PP session, we selected the following four time points based on the protocol described in a previously published study by another group: (1) supine before PP (just before the patient was turned to the PP); (2) during PP (4 h within the first PP); (3) end PP (just before the patient was turned back to the supine position); and (4) supine after PP (4 h after resupination) [22].

At each time point, ventilatory variables, arterial blood gas, hemodynamic status, and EIT data were collected. Due to the limited feasibility of obtaining high-quality echocardiographic images while patients were in PP, transthoracic echocardiography was performed only in the supine position before and after PP.

### EIT data analysis

EIT data were acquired using a standard device (PulmoVista^®^500; Dräger, Lübeck, Germany) at a sampling rate of 20 Hz and stored for offline analysis. The EIT belt was positioned directly below the armpits, between the fourth and fifth intercostal spaces. The position of the EIT belt was marked with a pen to avoid belt displacement after turning patients prone. After a 5-minute baseline recording of EIT data, an end-expiratory breath hold lasting 10 s was performed. Two seconds into the occlusion, a 10 mL bolus of 5% NaCl solution was manually injected through the central venous catheter over less than 2 s. The saline bolus traversed the pulmonary circulation, producing an impedance dilution curve that exhibited typical first-pass kinetics [23].

EIT ventilation maps were analyzed offline by averaging the values of five consecutive respiratory cycles selected from a visually stable segment within the 1-minute recording prior to saline injection, after respiratory and hemodynamic stabilization. For the quantitative analysis of ventilation and perfusion distributions, the lungs were divided into four non-overlapping horizontal regions of interest (ROIs) from ventral to dorsal: ventral (ROI 1), medial-ventral (ROI 2), medial-dorsal (ROI 3), and dorsal (ROI 4). Each ROI had an identical vertical height corresponding to 25% of the anteroposterior diameter.

The regional functional ventilation map was calculated by subtracting the end-expiratory impedance from the end-inspiratory impedance, representing the local volume variation during tidal breathing. From the ventilation map analysis, the following measures were assessed:

1. The percentage of ventilation distribution in each region was calculated.
2. The global inhomogeneity (GI) index was used to evaluate ventilation distribution [24].
3. The center of ventilation (CoV) was calculated to describe the vertical (ventral-to-dorsal) distribution [25].

Regional functional perfusion maps were derived by analyzing the slope of the time-impedance curve after saline injection, after excluding the cardiac region from the images. Ventilated and perfused regions were defined as pixels exceeding 20% of maximum values in the functional ventilation and perfusion maps, respectively. From the perfusion map analysis, four measures were calculated:

1. The percentage of perfusion distribution in each ROI.
2. Dead space (%): regions ventilated but not perfused.
3. Shunt (%): regions perfused but not ventilated.
4. V/Q matching (%): regions both ventilated and perfused.

### Prone positioning

In our respiratory ICU, PP is routinely performed for ARDS patients with PaO_2_/FiO_2_ < 150 mm Hg, with the final decision left to the treating physicians. Patients began PP within 24 h of enrollment and were maintained in that position for 16 consecutive hours. The criteria for stopping PP were as follows: (1) PaO_2_/FiO_2_ ≥ 150 mm Hg in the supine position 4 h after the end of PP; (2) a decrease in PaO_2_/FiO_2_ > 20% after PP compared with the ratio in the supine position; or (3) any other life-threatening complication requiring discontinuation, as determined by the attending physician. We collected the duration and number of PP sessions, as well as complications associated with PP.

### Classifications

**Fig. 1:**
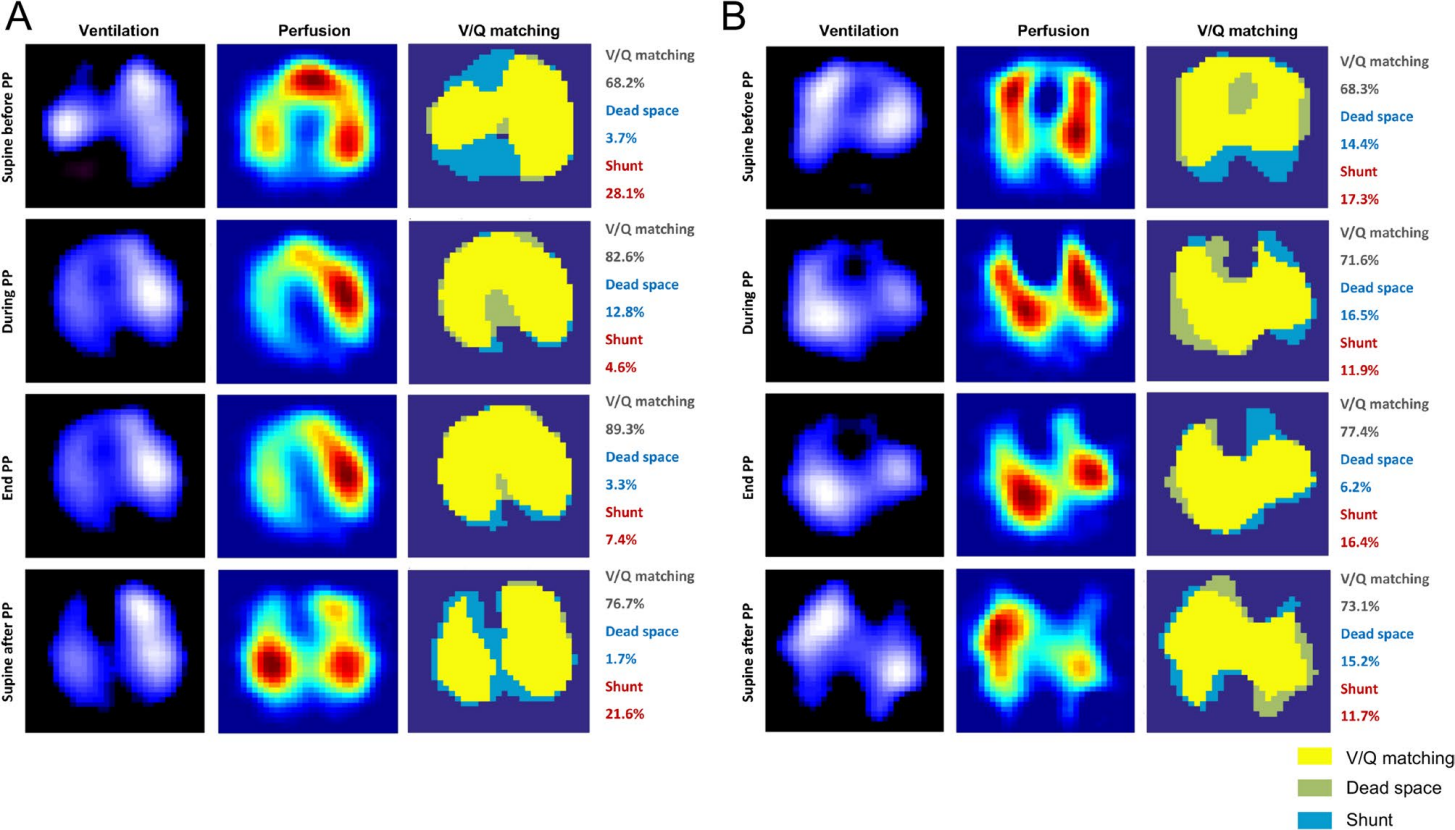
Changes in V/Q matching, dead space, and shunt during the first PP session in two representative patients with moderate to severe ARDS. Each row corresponds to different time points: supine before PP, during PP, end PP, and supine after PP. Quantitative percentages for each parameter are provided to the right of the V/Q matching maps. **(A)** A responder exhibited a significant improvement in V/Q matching, increasing from 68.2% before PP to 82.6% within 4 h of the first PP session. This was accompanied by a moderate increase in dead space from 3.7–12.8%, while shunt showed a substantial reduction, from 28.1–4.6%. **(B)** A non-responder showed minimal improvement in V/Q matching, with an increase from 68.3% before PP to 71.6% within 4 h of the first PP session. Dead space increased slightly, from 14.4–16.5%, while shunt decreased modestly, from 17.3–11.9%. V/Q ventilation-perfusion, PP prone positioning, ARDS, acute respiratory distress syndrome

Previous studies by other groups have shown that V/Q matching generally improves by about 10% in the early phase after PP [19, 20]. Therefore, patients in this study were classified as responders or non-responders based on whether their V/Q matching improvement reached ≥ 10% 4 h within the first PP (Fig. 1).

### Study endpoints

The primary endpoint was ICU mortality, while the secondary endpoint was ventilator-free days at day 28. Other endpoints recorded for each group included 28-day mortality, length of ICU and hospital stay, oxygenation deterioration requiring VV-ECMO support, use of continuous renal replacement therapy (CRRT), and tracheostomy. Information on the causes of death, including the presence of therapeutic limitations, was also collected.

### Statistical analysis

To calculate the study sample size, we assumed a 2:1 ratio between responders and non-responders. A total of 69 patients were required to detect a 20% difference in mortality between the two groups, with a power of 0.8 and an alpha level of 0.025. To account for potential dropouts, we included 77 patients in the study.

Continuous variables are presented as means ± standard deviation or medians with interquartile ranges. Comparisons between groups were performed using Student’s t-test or the Mann-Whitney U test, as appropriate. Categorical variables were analyzed using the chi-square test or Fisher’s exact test. Repeated measures analysis of variance (ANOVA) or the Friedman test, with post-hoc Bonferroni or Dunn’s multiple comparisons, were used to analyze data obtained at each time point during PP, when applicable. Correlation between continuous variables was assessed using the Spearman regression coefficient. ICU mortality was compared using Kaplan-Meier survival estimates, and a log-rank test was applied to compare the two groups. Risk factors associated with ICU mortality were identified through univariate analysis. Variables with statistical significance (*P* ≤ 0.1) in the univariate analysis were included in the multivariate analysis, which was conducted using multiple logistic regression with backward stepwise selection. No imputation for missing data was performed because all analyzed cases were complete.

All *P* values were two-sided, and values less than 0.05 were considered significant. Data were analyzed using R version 4.3.1 (R Foundation for Statistical Computing, Vienna, Austria) and GraphPad Prism version 8 (GraphPad Software Inc., San Diego, CA, USA).

## Results

### Study population

From April 2023 to October 2024, 77 patients were enrolled in the study, with 46 patients (59.7%) classified as responders and 31 patients (40.3%) as non-responders based on V/Q matching improvement of ≥ 10% 4 h within the first PP session (Figure S1). The main characteristics of the study population are summarized in Table 1. The mean age of patients was 61.5 ± 13.9 years, and 50 patients (64.9%) were male. The primary cause of ARDS was pneumonia (58.4%), with no significant difference in etiology between the groups. The proportion of severe ARDS was slightly higher in responders than in non-responders, but the difference was not statistically significant (41.3% vs. 29.0%; *P* = 0.272). The median duration of IMV before the first PP session was 7 (range, 5–8) hours. Hemodynamic parameters, arterial blood gases, and ventilatory variables at enrollment showed no significant differences between the two groups.

**Table 1:** Main characteristics of study population

Characteristic	All patients (*n* = 77)	Responders (*n* = 46)	Non-responders (*n* = 31)	*P*
**Age**,** years**	61.5 ± 13.9	60.4 ± 15.1	63.2 ± 12.0	0.428
**Male**,** no. (%)**	50 (64.9)	30 (65.2)	20 (64.5)	0.950
**Body mass index**,** kg/m**^**2**^	25.3 (23.4–27.1)	25.7 (23.5–27.2)	24.5 (23.2–27.6)	0.464
**APACHE II score at enrollment**	17 (13–22)	17 (13–23)	17 (11–22)	0.960
**SOFA score at enrollment**	10 (8–12)	9 (8–11)	11 (8–12)	0.358
**Murray score at enrollment**	3.00 (2.75–3.25)	3.00 (3.00–3.25)	3.25 (2.50–3.25)	0.877
**Cause of ARDS**,** no. (%)**				
Pneumonia	45 (58.4)	24 (52.2)	21 (71.0)	0.174
COVID-19 pneumonia	15 (19.5)	8 (17.4)	7 (22.6)	0.573
Aspiration	10 (13.0)	7 (15.2)	3 (9.7)	0.716
Non-pulmonary sepsis	12 (15.6)	9 (19.6)	3 (9.7)	0.394
Trauma	7 (9.1)	4 (8.7)	3 (9.7)	1.000
Acute pancreatitis	3 (3.9)	2 (4.3)	1 (3.2)	1.000
**ARDS severity**,** no. (%)**				0.272
Moderate: PaO_2_:FiO_2_ 100–200 mmHg	49 (63.6)	27 (58.7)	22 (71.0)	
Severe: PaO_2_:FiO_2_ < 100 mmHg	28 (36.4)	19 (41.3)	9 (29.0)	
**Comorbidity**,** no. (%)**				
Immunocompromised	14 (18.2)	10 (21.7)	4 (12.9)	0.324
COPD or asthma	15 (19.5)	10 (21.7)	5 (16.1)	0.542
Coronary artery disease	8 (10.4)	4 (8.7)	4 (12.9)	0.707
Hypertension	32 (41.6)	17 (37.0)	15 (48.4)	0.318
Diabetes mellitus	9 (11.7)	4 (8.7)	5 (16.1)	0.526
Chronic renal insufficiency	10 (13.0)	7 (15.2)	3 (9.7)	0.716
**Duration of IMV before first PP**,** hours**	7 (5–8)	6 (5–8)	7 (6–8)	0.423
**Hemodynamic status at enrollment**				
Norepinephrine dose, µg/kg/min	0.00 (0.00–0.30)	0.00 (0.00–0.30)	0.00 (0.00–0.35)	0.962
Lactate, mmol/L	1.7 (1.1–2.1)	1.7 (1.1–1.9)	1.7 (1.1–2.4)	0.396
Heart rate, beats/min	87.9 ± 21.2	87.4 ± 21.6	86.6 ± 21.1	0.817
Mean arterial pressure, mmHg	83.8 ± 11.2	84.8 ± 11.8	82.4 ± 10.4	0.414
CVP, mmHg	10 (7–13)	10 (7–13)	10 (8–14)	0.779
ScvO_2_, %	59.8 ± 10.3	58.7 ± 10.6	61.5 ± 10.0	0.296
**Arterial blood gas at enrollment**				
pH	7.41 (7.35–7.46)	7.42 (7.35–7.47)	7.40 (7.34–7.43)	0.211
PaO_2_, mmHg	69.4 ± 10.9	68.2 ± 11.2	71.2 ± 10.5	0.297
PaCO_2_, mmHg	41.2 ± 10.3	40.9 ± 10.6	41.5 ± 10.2	0.828
HCO_3_^−^, mmol/L	25.9 ± 4.4	26.0 ± 3.5	25.9 ± 5.6	0.916
PaO_2_:FiO_2_ ratio, mmHg	110.9 ± 23.1	108.7 ± 24.9	114.2 ± 20.2	0.357
**Ventilatory variables at enrollment**				
FiO_2_	0.60 (0.50–0.80)	0.60 (0.50–0.80)	0.60 (0.50–0.75)	0.724
PEEP, mmH_2_O	10.4 ± 1.9	10.6 ± 2.0	10.1 ± 1.8	0.306
Tidal volume, ml	415 ± 51	408 ± 52	425 ± 50	0.201
Tidal volume, ml/PBM	6.5 ± 0.7	6.4 ± 0.6	6.5 ± 0.9	0.623
Respiratory rate, breaths/min	23.9 ± 3.0	24.1 ± 3.2	23.5 ± 2.7	0.430
Peak airway pressure, cmH_2_O	27.1 ± 4.5	27.7 ± 5.0	26.1 ± 3.5	0.156
Plateau pressure, cmH_2_O	22.8 ± 4.0	23.2 ± 4.1	22.2 ± 3.8	0.296
Driving pressure, cmH_2_O	12.4 ± 3.2	12.7 ± 3.3	12.1 ± 3.1	0.499
Compliance, ml/cmH_2_O	35.2 ± 8.0	34.1 ± 8.5	36.8 ± 7.0	0.197

APACHE II Acute Physiology and Chronic Health Evaluation II, ICU intensive care unit, SOFA sequential organ failure assessment, ARDS acute respiratory distress syndrome, COVID-19 coronavirus disease 2019, PaO_2_:FiO_2_ ratio of the partial pressure of arterial oxygen to the fraction of inspired oxygen, COPD chronic obstructive pulmonary disease, IMV invasive mechanical ventilation, PP prone positioning, CVP central venous pressure, ScvO_2_ central venous oxygen saturation, PaO_2_ partial pressure of arterial oxygen, PaCO_2_ partial pressure of arterial carbon dioxide, HCO_3_^−^ bicarbonate, PEEP positive end-expiratory pressure, PBM predicted body weight

### Change in ventilatory variables, arterial blood gases, and hemodynamic status

FiO_2_ was significantly reduced during PP (*P* < 0.001). Plateau pressure and driving pressure progressively decreased (*P* < 0.001), while compliance significantly improved during PP and remained elevated in the supine position after PP (*P* < 0.001) (Table 2). PEEP, tidal volume, driving pressure, and compliance showed no significant differences between responders and non-responders at the four identified time points during the first PP session (Table S1 and Figure S2).

**Table 2:** Ventilatory variables, arterial blood gas, hemodynamic status, and electrical impedance tomography data at four identified time points throughout the first PP session

Variables	Supine before PP (*n* = 77)	During PP (*n* = 77)	End PP (*n* = 77)	Supine after PP (*n* = 77)	*P*
**Ventilatory variables**					
FiO_2_	0.60 (0.50–0.70)	0.50 (0.40–0.55)*	0.50 (0.40–0.55)*	0.50 (0.40–0.55)*	< 0.001
PEEP, mmH_2_O	10.6 ± 1.9	10.5 ± 2.1	10.4 ± 2.0	10.6 ± 1.8	0.331
Tidal volume, ml	416 ± 53	410 ± 57	414 ± 55	413 ± 52	0.771
Tidal volume, ml/PBM	6.5 ± 0.8	6.3 ± 0.8	6.4 ± 0.8	6.4 ± 0.8	0.800
Respiratory rate, breaths/min	23.6 ± 3.3	23.0 ± 4.0	22.4 ± 3.5	23.2 ± 3.6	0.174
Peak airway pressure, cmH_2_O	27.2 ± 5.0	27.2 ± 4.8	26.6 ± 4.7^#^	26.5 ± 4.5^#^	0.010
Plateau pressure, cmH_2_O	23.1 ± 4.3	22.5 ± 4.2^#^	21.9 ± 3.8*	22.3 ± 4.0*	< 0.001
Driving pressure, cmH_2_O	12.5 ± 3.5	12.0 ± 3.5^#^	11.4 ± 3.2*	11.7 ± 3.3*	< 0.001
Compliance, ml/cmH_2_O	35.4 ± 8.6	36.3 ± 8.4^#^	38.1 ± 8.9*	37.2 ± 8.6*	< 0.001
**Arterial blood gas**					
pH	7.42 (7.37–7.45)	7.43 (7.38–7.47)^#^	7.43 (7.39–7.47)^#^	7.42 (7.37–7.45)	0.001
PaO_2_, mmHg	69.1 ± 11.7	78.0 ± 9.8*	83.5 ± 10.6*	79.2 ± 7.9*	< 0.001
PaCO_2_, mmHg	41.8 ± 9.7	40.4 ± 10.0*	39.6 ± 9.9*	40.5 ± 10.0*	< 0.001
HCO_3_^−^, mmol/L	25.9 ± 4.4	26.0 ± 3.9	25.6 ± 3.8	26.0 ± 3.4	0.621
PaO_2_:FiO_2_ ratio, mmHg	113.2 ± 24.9	162.0 ± 27.1*	178.4 ± 36.8*	162.8 ± 31.3*	< 0.001
Ventilatory ratio	1.59 (1.33–1.88)	1.48 (1.26–1.94)*	1.42 (1.16–1.80)*	1.50 (1.24–1.87)^#^	< 0.001
**Hemodynamic status**					
Norepinephrine dose, µg/kg/min	0.00 (0.00–0.30)	0.05 (0.00–0.30)	0.05 (0.05–0.30)	0.05 (0.00–0.30)	0.219
Lactate, mmol/L	1.5 (0.9–2.2)	1.4 (1.0–1.9)	1.4 (0.9–2.2)	1.3 (1.0–1.9)	0.412
Heart rate, beats/min	92.1 ± 17.6	93.3 ± 20.3	91.4 ± 17.5	88.3 ± 17.5	0.072
Mean arterial pressure, mmHg	81.4 ± 12.9	80.6 ± 12.4	80.3 ± 10.9	83.7 ± 9.9	0.193
CVP, mmHg	10 (7–13)	12 (10–14)^#^	13 (10–14)*	11 (8–12)	< 0.001
ScvO_2_, %	59.5 ± 11.4	67.3 ± 9.6*	71.6 ± 11.3*	67.3 ± 8.0*	< 0.001
**Electrical impedance tomography**					
Ventilation distribution, ventral, %	63.0 ± 14.9	45.5 ± 15.1*	41.5 ± 13.8*	53.5 ± 13.3*	< 0.001
Ventilation distribution, dorsal, %	37.0 ± 14.9	54.5 ± 15.1*	58.5 ± 13.8*	46.5 ± 13.3*	< 0.001
Ventilation distribution, ROI 1, %	12.0 (8.0–19.5)	7.0 (4.6–12.1)*	8.0 (5.3–10.4)*	9.6 (5.4–14.1)*	< 0.001
Ventilation distribution, ROI 2, %	49.0 ± 11.8	36.7 ± 11.4*	33.5 ± 10.4*	42.6 ± 9.5*	< 0.001
Ventilation distribution, ROI 3, %	30.6 ± 13.1	44.6 ± 12.2*	47.1 ± 10.0*	37.9 ± 13.4*	< 0.001
Ventilation distribution, ROI 4, %	5.7 (3.8–8.4)	7.4 (5.0–13.9)^#^	9.4 (6.3–13.2)*	8.0 (3.9–12.7)	0.002
GI index ventilation	0.59 (0.56–0.64)	0.52 (0.50–0.54)*	0.50 (0.47–0.52)*	0.52 (0.49–0.54)*	< 0.001
Center of ventilation, %	44.19 ± 5.77	51.24 ± 5.38*	52.59 ± 8.41*	47.80 ± 5.92*	< 0.001
Perfusion distribution, ventral, %	58.8 ± 11.3	42.6 ± 9.1*	41.1 ± 9.2*	50.4 ± 4.3*	< 0.001
Perfusion distribution, dorsal, %	41.2 ± 11.3	57.4 ± 9.1*	58.9 ± 9.2*	49.6 ± 4.3*	< 0.001
Perfusion distribution, ROI 1, %	15.6 ± 6.5	9.1 ± 3.8*	9.0 ± 4.4*	13.4 ± 2.6^#^	< 0.001
Perfusion distribution, ROI 2, %	43.3 ± 9.0	33.5 ± 7.7*	32.1 ± 7.5*	37.0 ± 3.5*	< 0.001
Perfusion distribution, ROI 3, %	31.5 ± 9.3	43.7 ± 8.7*	44.7 ± 8.2*	39.0 ± 3.2*	< 0.001
Perfusion distribution, ROI 4, %	7.5 (4.9–12.5)	12.8 (7.5–20.4)^#^	13.2 (8.4–19.1)*	10.5 (6.9–13.3)	0.003
Shunt, %	22.1 ± 9.6	17.8 ± 9.8*	17.2 ± 10.1*	19.1 ± 11.3	0.008
Shunt, ventral, %	6.9 ± 3.0	6.7 ± 3.6	6.8 ± 3.8	6.5 ± 3.9	0.901
Shunt, dorsal, %	15.2 ± 6.6	11.0 ± 6.3*	10.4 ± 6.4*	12.6 ± 7.5^#^	< 0.001
Dead space, %	23.1 ± 10.7	15.4 ± 7.2*	13.5 ± 8.9*	19.1 ± 12.2^#^	< 0.001
Dead space, ventral, %	17.3 ± 8.1	10.1 ± 4.7*	8.7 ± 5.9*	14.0 ± 9.1^#^	< 0.001
Dead space, dorsal, %	5.7 ± 2.7	5.2 ± 2.6	4.8 ± 3.1^#^	5.1 ± 3.3	0.208
V/Q matching, %	59.0 ± 8.6	71.2 ± 7.8*	71.6 ± 10.9*	61.8 ± 12.9	< 0.001
V/Q matching, ventral, %	33.9 ± 5.3	38.0 ± 4.5*	35.7 ± 5.6^#^	34.1 ± 7.1	< 0.001
V/Q matching, dorsal, %	25.1 ± 3.9	33.2 ± 4.4*	35.8 ± 6.0*	27.7 ± 6.3*	< 0.001

PP prone positioning, FiO_2_ the fraction of inspired oxygen, PEEP positive end-expiratory pressure, PBM predicted body weight, PaO_2_ partial pressure of arterial oxygen, PaCO_2_ partial pressure of arterial carbon dioxide, HCO_3_^−^ bicarbonate, CVP central venous pressure, ScvO_2_ central venous oxygen saturation, ROI region of interest, GI, global inhomogeneity, V/Q ventilation/perfusion

*P* value by one-way analysis of variance (ANOVA) for repeated measures

^#^*P <* 0.05, * *P <* 0.01 compare with before PP

Arterial blood gas analysis showed significant increases in PaO_2_ and PaO_2_/FiO_2_ during PP, with sustained improvement after PP (*P* < 0.001). Partial pressure of arterial carbon dioxide (PaCO_2_) levels decreased significantly during PP and remained lower in the supine position after PP (*P* < 0.001) (Table 2). Responders demonstrated significantly higher PaO_2_/FiO_2_ than non-responders during PP, at the end of PP, and in the supine position after PP (*P* < 0.001) (Table S1 and Figure S3).

Hemodynamic parameters were stable throughout the first PP session. No significant changes were noted in norepinephrine doses, lactate levels, or mean arterial pressure across the four time points. Heart rate exhibited a slight but non-significant decrease from the supine position before PP to the supine after PP (*P* = 0.072). Both central venous pressure (CVP) and central venous oxygen saturation (ScvO_2_) increased significantly during PP, with ScvO_2_ remaining elevated even after resupination (*P* < 0.001) (Table 2, Table S1, and Figure S4).

### Change in ventilation, perfusion, and V/Q matching

**Fig. 2:**
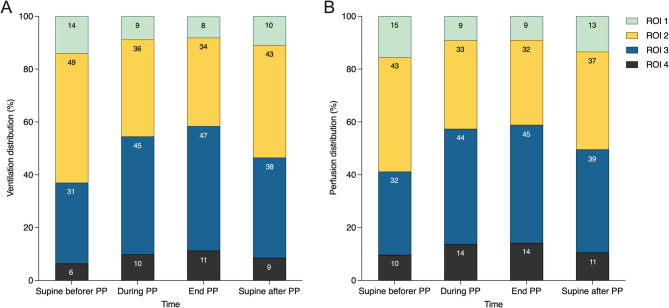
Changes in ventilation **(A)** and perfusion **(B)** distribution across four ventral-to-dorsal horizontal regions during the first PP session. PP prone positioning

EIT data revealed a redistribution of ventilation and perfusion from ventral to dorsal regions during PP (*P* < 0.001) (Table 2; Fig. 2, and Figure S5).

**Fig. 3:**
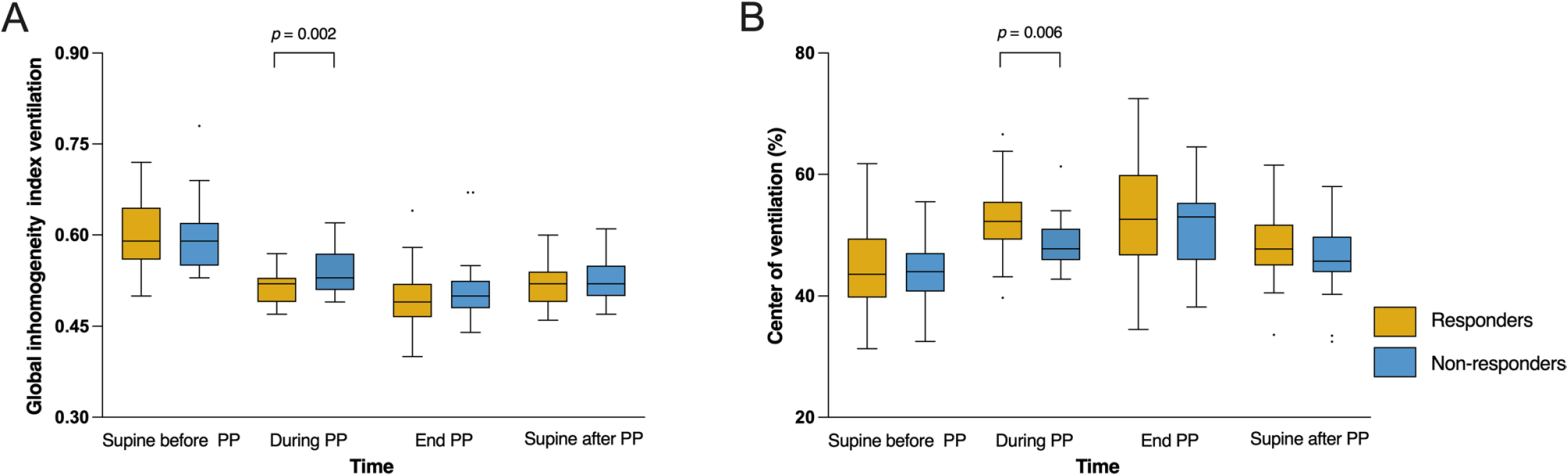
Comparison of global inhomogeneity index **(A)** and center of ventilation **(B)** between responders and non-responders during the first PP session. PP prone positioning

The GI index decreased significantly during PP (*P* < 0.001), and this reduction was sustained in the supine position after PP. The CoV shifted significantly toward the dorsal regions during PP (*P* < 0.001), with this shift partially persisting in the supine position after PP. The GI index was significantly lower in responders than in non-responders (*P* = 0.018), while the CoV was significantly higher in responders than in non-responders (*P* = 0.006) during PP (Table S1 and Fig. 3).

**Fig. 4:**
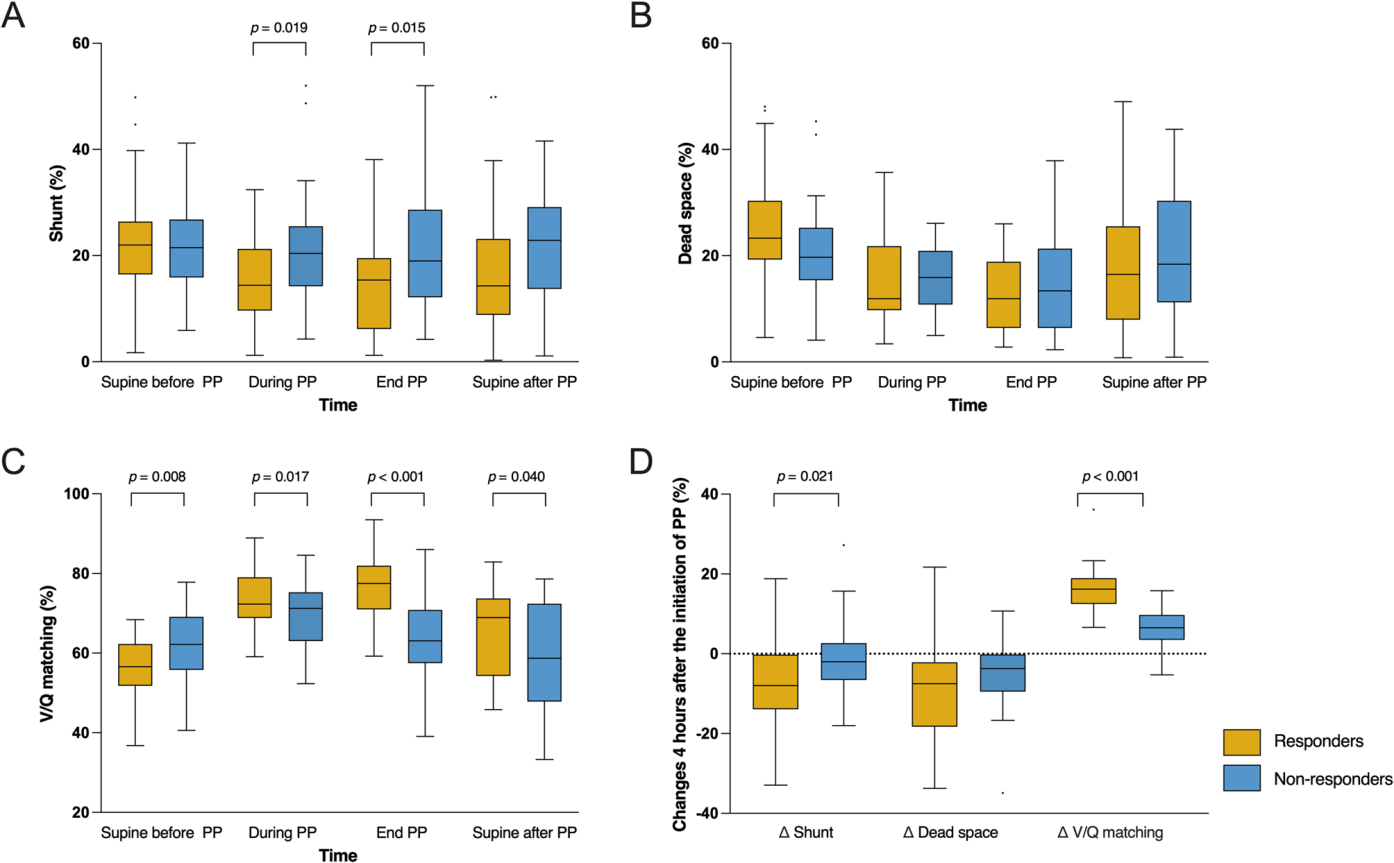
Comparison of shunt **(A)**, dead space **(B)**, V/Q matching **(C)**, and their respective changes 4 h after the initiation of PP **(D)** between responders and non-responders during the first PP session. V/Q ventilation-perfusion, PP prone positioning

Shunt decreased significantly during PP (*P* = 0.008), with the reduction predominantly in the dorsal region (*P* < 0.001). Similarly, dead space decreased significantly (*P* < 0.001), primarily in the ventral region (*P* < 0.001). V/Q matching improved significantly in the first 4 h of PP, increasing from 59.0 ± 8.6% before PP to 71.2 ± 7.8% (*P* < 0.001). However, no further significant improvement was observed by the end of PP, and improvement was partially sustained in the supine position after PP (59.0 ± 8.6% vs. 61.8 ± 12.9%; *P* = 0.084) (Table 2). Responders showed significantly lower shunt than non-responders during PP (*P* = 0.019) and at the end of PP (*P* = 0.015). V/Q matching was significantly higher in responders during PP (*P* = 0.017), at the end of PP (*P* < 0.001), and in the supine position after PP (*P* = 0.040). Furthermore, the improvement in V/Q matching in responders was significantly superior to that of non-responders 4 h within the first PP (*P* < 0.001) (Table S1 and Fig. 4).

**Fig. 5:**
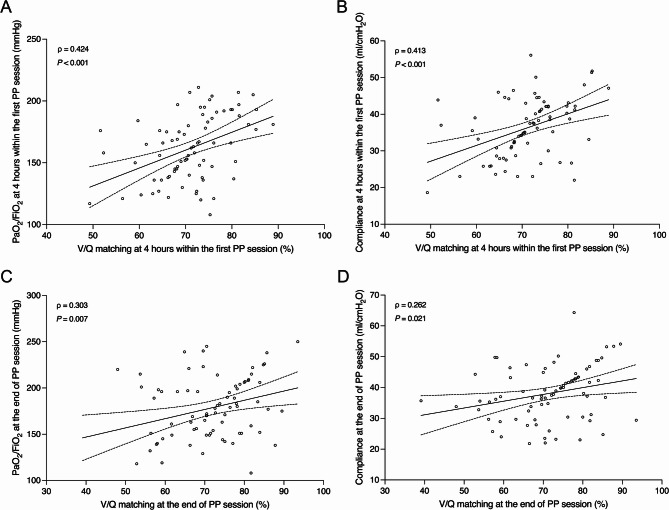
Relationship between V/Q matching and PaO_2_/FiO_2_ at 4 h within the first PP session **(A)**, V/Q matching and respiratory system compliance at 4 h within the first PP session **(B)**, V/Q matching and PaO_2_/FiO_2_ at the end of PP session **(C)**, and V/Q matching and respiratory system compliance at the end of PP session **(D)** V/Q ventilation-perfusion, PaO_2_/FiO_2_ ratio of the partial pressure of arterial oxygen to the fraction of inspired oxygen, PP prone positioning

V/Q matching at 4 h within the first PP session was positively correlated with PaO_2_/FiO_2_ (ρ = 0.424, *P* < 0.001) and respiratory system compliance (ρ = 0.413, *P* < 0.001). At the end of PP session, V/Q matching remained moderately associated with PaO_2_/FiO_2_ (ρ = 0.303, *P* = 0.007) and respiratory system compliance (ρ = 0.262, *P* = 0.021) (Fig. 5).

### Clinical outcomes, PP-related complications, and cause of death

**Fig. 6:**
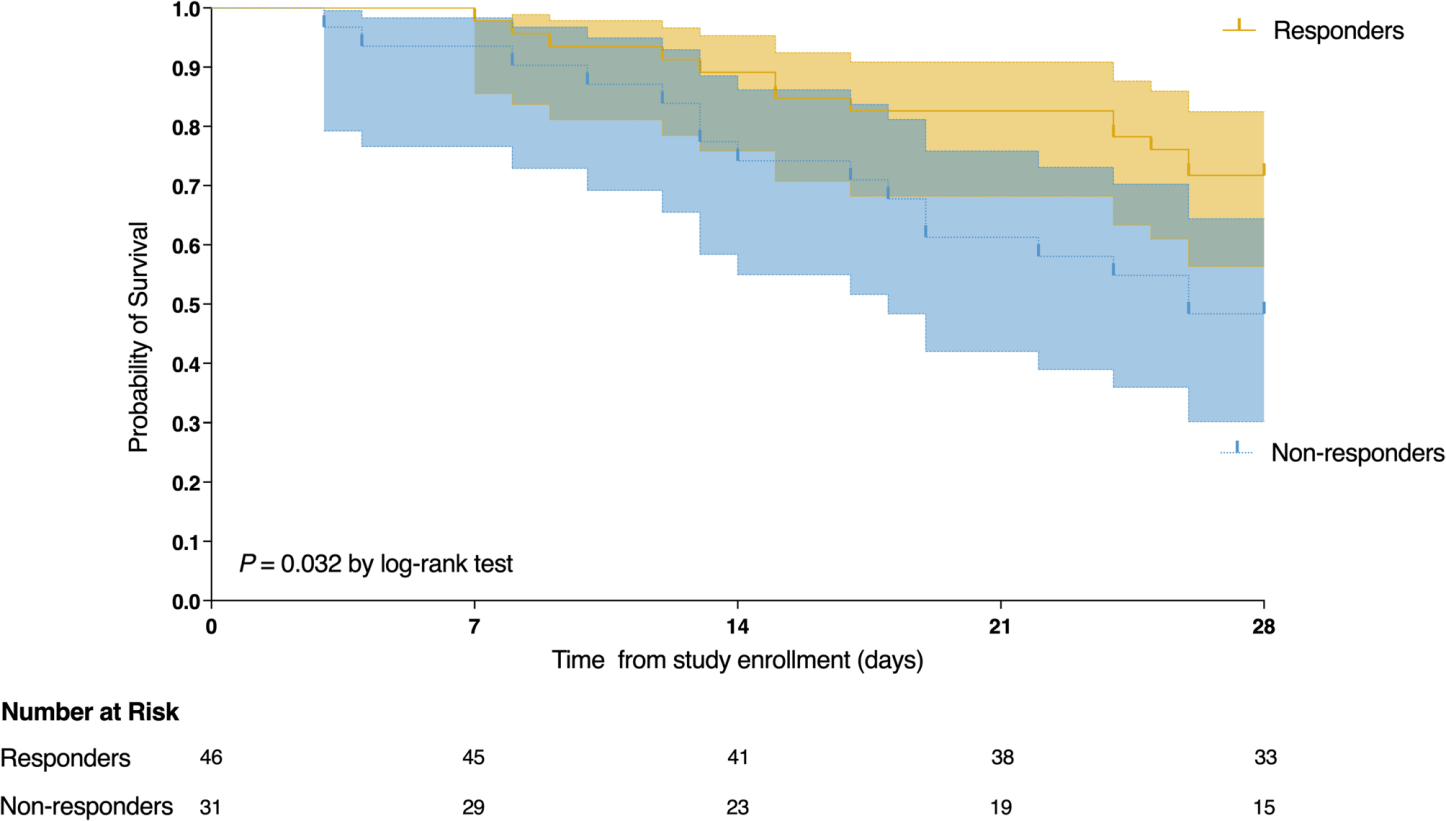
Probability of survival from day of study enrollment to day 28 in responders and non-responders

Clinical outcomes, PP-related complications, and cause of death are shown in Table 3. ICU mortality and 28-day mortality was significantly lower in responders than in non-responders (28.3% vs. 51.6%; *P* = 0.038). Kaplan-Meier analysis from study enrollment to day 28 further demonstrated that responders had a significantly lower ICU mortality than non-responders (*P* = 0.032) (Fig. 6). Ventilator-free days at day 28 were significantly higher in responders than in non-responders (16 [range, 0–21] days vs. 9 [range, 0–15] days; *P* = 0.024). Notably, three non-responders required VV-ECMO support due to oxygenation deterioration. There were no significant differences in ICU or hospital length of stay, CRRT, or tracheostomy between the groups.

**Table 3:** Clinical outcomes, PP-related complications, and cause of death

Outcome	All patients (*n* = 77)	Responders (*n* = 46)	Non-responders (*n* = 31)	*P*
**Primary end point**				
ICU mortality no. (%)	29 (37.7)	13 (28.3)	16 (51.6)	0.038
**Secondary end point**				
Ventilator-free days at day 28, days	13 (0–18)	16 (0–21)	9 (0–15)	0.024
**Other end points**				
28-day mortality, no. (%)	29 (37.7)	13 (28.3)	16 (51.6)	0.038
ICU length of stay, days	18 (10–26)	18 (11–25)	19 (10–27)	0.615
Hospital length of stay, days	24 (14–33)	24 (16–32)	21 (13–35)	0.656
Veno-venous ECMO, no. (%)	4 (5.2)	0 (0.0)	3 (9.7)	0.061
CRRT, no. (%)	17 (22.1)	11 (23.9)	6 (19.4)	0.636
Tracheostomy, no. (%)	23 (29.9)	13 (28.3)	10 (32.3)	0.707
**Prone positioning**				
Duration of first PP, hours	16 (15–16)	16 (14–16)	16 (15–16)	0.196
Number of PP sessions	3 (2–5)	3 (3–5)	3 (2–5)	0.339
Major complications no. (%)				
Accidental extubation	1 (1.3)	1 (2.2)	0 (0.0)	1.000
Airway dislodgement	5 (6.5)	3 (6.5)	2 (6.5)	1.000
Endotracheal tube obstruction	5 (6.5)	2 (4.3)	3 (9.7)	0.387
Minor complications no. (%)				
Hemodynamic instability	4 (5.2)	2 (4.3)	2 (6.5)	1.000
Vomiting	6 (7.8)	3 (6.5)	3 (9.7)	0.680
Facial swelling	12 (15.6)	8 (17.4)	4 (12.9)	0.832
**Cause of death**,** no. (%)**				
Sepsis	10 (13.0)	4 (8.7)	6 (19.4)	0.172
Pulmonary	5 (6.5)	2 (4.3)	3 (9.7)	0.387
Cardiac	7 (9.1)	4 (8.7)	3 (9.7)	1.000
Hepatic	3 (3.9)	1 (2.2)	2 (6.5)	0.561
Hemorrhage	2 (2.6)	1 (2.2)	1 (3.2)	1.000
Therapeutic limitations	2 (2.6)	1 (2.2)	1 (3.2)	1.000

PP prone positioning, PaO_2_:FiO_2_ ratio of the partial pressure of arterial oxygen to the fraction of inspired oxygen, PP prone positioning, ICU intensive care unit, ECMO extracorporeal membrane oxygenation, CRRT continuous renal replacement therapy

The length of the first PP session was similar between responders and non-responders (16 [range, 14–16] hours vs. 16 [range, 14–16] hours; *P* = 0.196). The number of PP sessions per patient was also comparable between the two groups (3 [range, 3–5] vs. 3 [range, 2–5]; *P* = 0.339). In addition, there were no significant differences in PP- related complications between responders and non-responders.

The causes of death did not differ significantly between responders and non-responders. One patient in each group died after therapeutic limitations.

### Risk factors associated with ICU mortality

Univariate analysis identified age, APACHE II scores, pre-PP driving pressure, and changes in post-PP driving pressure, PaO_2_/FiO_2_, compliance, shunt, dead space, and V/Q matching as significant predictors of ICU mortality (Table 4).

**Table 4:** Risk factors associated with ICU mortality

Variables	Univariate logistic regression			Multivariate logistic regression		
	Odds ratios	95% CI	*P*	Odds ratios	95% CI	*P*
**Study center**	1.036	0.370–2.907	0.946			
**Pre-PP**						
Age	1.063	1.016–1.112	0.008			
Body mass index	1.037	0.862–1.247	0.701			
APACHE II score	1.090	1.003–1.185	0.043			
SOFA score	1.205	0.989–1.468	0.064			
ARDS severity	2.333	0.796–6.837	0.122			
Immunocompromised	3.828	0.979–14.967	0.054			
Lactate	1.166	0.679–2.001	0.578			
PaO_2_:FiO_2_ ratio	0.980	0.957–1.003	0.086			
Driving pressure	1.262	1.052–1.514	0.012	1.473	1.132–1.916	0.004
Compliance	0.941	0.878–1.008	0.082			
**Post-4-hour PP changes**						
Δ Driving pressure	2.016	1.201–3.387	0.008	2.919	1.366–6.236	0.006
Δ Compliance	0.770	0.621–0.954	0.017			
Δ PaCO_2_	1.068	0.761–1.498	0.704			
Δ PaO_2_:FiO_2_ ratio	0.982	0.965–0.999	0.033			
Δ Shunt	1.061	1.004–1.121	0.037			
Δ Dead space	1.082	1.014–1.155	0.017			
Δ V/Q matching	0.832	0.745–0.930	0.001	0.790	0.681–0.917	0.002

PP prone positioning, APACHE II Acute Physiology and Chronic Health Evaluation II, SOFA sequential organ failure assessment, PaO_2_:FiO_2_ ratio of the partial pressure of arterial oxygen to the fraction of inspired oxygen, Δ changes after 4 h of prone positioning, PaCO_2_ partial pressure of arterial carbon dioxide, V/Q ventilation/perfusion

Multivariate analysis confirmed that improved V/Q matching post-PP was protective against mortality (OR, 0.790; 95% CI, 0.681–0.917; *P* = 0.002). Meanwhile, higher pre-PP driving pressure (OR, 1.473; 95% CI, 1.132–1.916; *P* = 0.004) and increased post-PP driving pressure (OR, 2.919; 95% CI, 1.366–2.236; *P* = 0.006) were associated with higher mortality (Table 4).

## Discussion

To our knowledge, this is the first prospective observational study to explore the relationship between V/Q matching improvements following initial PP and ICU mortality in patients with moderate to severe ARDS. Through the use of EIT, we observed that: (1) PP led to significant enhancements in V/Q matching by reducing dorsal shunt and ventral dead space, with partial effects sustained after returning to the supine position; (2) responders showed lower ICU and 28-day mortality, more ventilator-free days at 28 days, and a reduced need for VV-ECMO than non-responders; (3) responders achieved greater physiological improvements, including higher PaO_2_/FiO_2_ and more substantial reductions in shunt during PP than non-responders; and (4) enhanced V/Q matching was independently associated with lower ICU mortality.

PP enhances V/Q matching by reducing shunt in the dorsal regions and minimizing dead space in the ventral regions [26]. Our study found that this redistribution of ventilation and perfusion promotes alveolar recruitment in the well-perfused dorsal areas while relieving overdistension in the less-perfused ventral regions, resulting in an overall improvement in V/Q matching. These results are consistent with previous studies that demonstrated significant enhancements in V/Q matching with PP, highlighting its effectiveness in reducing the impact of pleural pressure gradient on ventilation and perfusion [17–20]. While most studies have focused on changes in V/Q matching during the initial phase of PP [17, 18, 20], it is important to note that the effect of PP is time dependent [27]. Our study addressed this limitation by monitoring changes in ventilation and perfusion throughout the prone session and after resupination. Notably, the improvement in V/Q matching observed during PP was partially maintained in the supine position, particularly in the dorsal regions. These findings contribute to a deeper understanding of PP as a dynamic and sustained intervention for optimizing V/Q matching in ARDS.

The selection of 4 h within the first PP as the time point for defining responders and non-responders was based on a previous study and our clinical observations [22]. That study reported the most pronounced PaO_2_/FiO_2_ improvement within the first 4 h of PP, followed by a more gradual increase. Similarly, our study found that PaO_2_/FiO_2_ rose from 113.2 mmHg to 162.0 mmHg within the first 4 h of PP, then increased more slowly, reaching 178.4 mmHg by the end of PP. Since PaO_2_/FiO_2_ improvement largely depends on V/Q matching enhancement [19], we hypothesized that the primary improvement in V/Q matching would also occur within the first 4 h of PP. Our findings confirmed this hypothesis, showing that V/Q matching improved predominantly within the first 4 h of PP, rising from 59.0% before PP to 71.2% at 4 h, with minimal further change thereafter, reaching 71.6% by the end of PP. Moreover, using the end of the PP session as the defining time point would introduce variability, as the duration of PP differed among patients. Therefore, selecting a fixed 4-hour time point ensured consistency and comparability across the study population.

However, despite this general trend, the degree of V/Q matching improvement varied considerably among patients, indicating that not all individuals benefited equally from PP [28]. In this study, 59.7% (46/77) of patients experienced a V/Q matching improvement greater than 10% from PP. Several factors may contribute to this heterogeneity. First, the etiology of ARDS plays a critical role. Patients with extrapulmonary ARDS often respond better to PP than those with pulmonary ARDS [29]. This difference may be explained by the ability of PP to reverse compressive atelectasis in extrapulmonary ARDS, while its effects on consolidated lung units in pulmonary ARDS are more limited and less immediate [30]. In our study, extrapulmonary ARDS was slightly more common among responders than non-responders (32.6% vs. 22.6%; *P* = 0.339), suggesting a possible association between ARDS type and V/Q matching improvement. Second, the severity of illness may influence the response to PP. In more severe cases, reduced hypoxic vasoconstriction in the dorsal lung regions after oxygenation improvement could lead to a more pronounced decrease in dorsal shunt, resulting in greater V/Q matching improvement [31]. This is further supported by our finding that moderate to severe ARDS was more frequently observed in responders than in non-responders (41.3% vs. 29.0%; *P* = 0.272). Finally, the timing of PP initiation is a critical factor. A previous study showed that PP significantly reduces V/Q mismatch in patients with early ARDS but may worsen V/Q mismatch in those with persistent ARDS [32]. In our study, all patients underwent PP shortly after the start of IMV, ensuring they were in the early phase of ARDS. As a result, most patients showed improvements in V/Q matching after PP, although the degree of improvement varied widely among patients.

Our study found that responders had significantly lower ICU mortality than non-responders. This difference may be attributed to the larger areas of unmatched ventilation and perfusion observed in non-responders during PP, which could increase the risk of VILI. Non-responders often require higher pressures to recruit collapsed lung regions to reduce shunt [33]. Simultaneously, increased minute ventilation is needed to compensate for alveolar hypoventilation caused by elevated dead space [34]. These mechanical demands place significant stress and strain on the lungs, heightening the risk of overdistension, repetitive alveolar opening and closing, and the development of VILI [35, 36]. Furthermore, responders exhibited significantly higher PaO₂/FiO₂ ratios than non-responders, a difference that persisted from PP to the period after resupination, potentially contributing to improved organ oxygen delivery. A previous study demonstrated that the percentage of unmatched lung units is an independent predictor of mortality in ARDS patients, consistent with our findings [37]. This further supports the hypothesis that PP reduces mortality by improving V/Q matching and alleviating the physiological burden of ARDS.

In this study, we found that PP reduced driving pressure and improved respiratory system compliance, with these effects becoming more evident as PP duration increased. Furthermore, the results of the GI index and the CoV confirmed that lung ventilation became more homogenous during PP. Previous studies have not consistently observed improvements in respiratory system compliance during PP [17, 20]. A possible explanation is the relatively short duration of PP in those studies. In the early phase of PP, the increase in lung compliance may be insufficient to fully counterbalance the decline in chest wall compliance, leading to minimal or no changes in overall respiratory system compliance [38]. However, studies with PP durations exceeding 12 h have shown significant improvements in respiratory system compliance compared with the supine position, aligning with our findings [19, 39]. Moreover, our study found that higher pre-PP driving pressure and increased post-PP driving pressure were associated with higher mortality, consistent with the findings of previous research [40, 41].

This study found that, similar to ventilation, perfusion also undergoes a ventral-to-dorsal shift during PP, contributing to maximal improvement in V/Q matching. However, it has been suggested that gravity has a minimal effect on pulmonary blood flow, with the gravitational distribution being only slightly altered by turning prone [42]. In the supine position, consolidation in the dependent dorsal regions leads to alveolar hypoxia, triggering hypoxic pulmonary vasoconstriction and redirecting blood flow to the better-ventilated nondependent ventral regions [43]. During PP, the redistribution of blood flow directs it toward the now-dependent dorsal regions, which receive better ventilation in this position. The previously described mechanism explains the shift of blood flow from the ventral to the dorsal regions observed in our study during PP.

We also observed that V/Q matching at 4 h within the first PP session was positively correlated with PaO_2_/FiO_2_ and respiratory system compliance, suggesting that improved V/Q matching may contribute to better oxygenation and lung mechanics. These correlations remained moderate at the end of the session, indicating a potential lasting physiological effect. This finding is consistent with previous studies showing that improved V/Q matching is associated with better oxygenation [19]. Since V/Q matching integrates data on ventilation and perfusion, it may provide complementary information to traditional respiratory parameters in identifying patients who are more likely to benefit from PP.

In this study, PEEP was set using a decremental titration after a recruitment maneuver to achieve the highest respiratory system compliance in the supine position, and it remained constant during PP. This approach aimed to avoid changes in ventilation and perfusion caused by PEEP adjustments, ensuring a precise evaluation of the impact of PP. Notably, PP and PEEP may have synergistic effects. By reducing the heterogeneity of regional lung strain, PP could mitigate the regional hyperinflation associated with high PEEP levels [4]. A recent study using EIT demonstrated that PEEP increases influenced V/Q matching through a balance between regional overdistension and recruitment [44]. This highlights the complex interplay between PP and PEEP in improving V/Q matching in ARDS patients.

The changes in V/Q matching observed during the first PP can help identify patients at greater risk for death, making it a valuable indicator for determining who may benefit from additional levels of assistance. Beyond PP, other interventions such as inhaled nitric oxide and VV-ECMO can also be considered to improve V/Q matching and oxygenation in ARDS patients [45, 46]. In our study, three non-responders required VV-ECMO therapy, underscoring the importance of alternative strategies for patients who do not respond to PP.

Beyond being the first study to explore the association between V/Q matching improvement with initial PP and ICU mortality in patients with moderate to severe ARDS, our study had several limitations. First, although this was a two-center prospective cohort study, the sample size was relatively small. We cannot exclude the possibility that the study was not adequately powered. Second, an EIT perfusion assessment using a saline bolus injection is still relatively new in clinical practice, although it has been validated in animal studies [13–15]. Therefore, these findings should be generalized with caution. Third, EIT has limited spatial resolution and evaluates only the lung areas enclosed by the belt, which may not represent the entire pulmonary parenchyma. Fourth, we did not measure esophageal pressure to distinguish chest wall compliance from lung compliance. This may have restricted our ability to accurately assess individual changes in chest wall and lung compliance during PP. Fifth, transthoracic echocardiography was performed only in the supine position before and after PP, which may have limited the assessment of cardiac function and its influence on V/Q matching during PP [47]. Last, as V/Q matching was assessed only during the first PP session, its ability to reflect the cumulative effect of multiple sessions remains unclear. Further studies are needed to evaluate whether responses to subsequent PP sessions could better predict outcomes.

## Conclusion

In conclusion, our study suggests that improvement in V/Q matching 4 h within the first PP session may be associated with lower ICU mortality in patients with moderate to severe ARDS. PP not only enhanced V/Q matching but also promoted more homogenous ventilation and perfusion, improving respiratory mechanics. These results underscore the importance of PP in managing ARDS and highlight the need for further research to develop personalized PP approaches and evaluate complementary treatments.

## Electronic supplementary material

Below is the link to the electronic supplementary material.

Supplementary Material 1: Additional file 1: 1. Online Table 1.1 Table S1. Comparison of ventilatory variables, arterial blood gas, hemodynamic status, and electrical impedance tomography between responder and non-responder groups during the first PP session. 1.2 Table S2 Changes in shunt, dead space, and V/Q matching 4 hours within the first PP in responders and non-responders. 1.3 Table S3 Comparison of echocardiographic variables between responders and non-responders in the supine position before and after PP. 2. Online Figures. 2.1 Figure S1 Flow chart. 2.2 Figure S2 Comparison of PEEP (A), tidal volume (B), driving pressure (C), and respiratory system compliance (D) between responders and non-responders during the first PP session. 2.3 Figure S3 Comparison of PaO_2_ (A), PaCO_2_ (B), PaO_2_/FiO_2_ (C), and ventilatory ratio (D) between responders and non-responders during the first PP session. 2.4 Figure S4 Comparison of CVP (A) and ScvO_2_ (B) between responders and non-responders during the first PP session. 2.5 Figure S5 Changes in ventilation (A) and perfusion (B) distribution across four ventral-to-dorsal horizontal regions during the first PP session.

Supplementary Material 2

## Data Availability

The data are available from the corresponding author on reasonable request.

## Abbreviations

PP: Prone positioning
ARDS: Acute respiratory distress syndrome
VILI: Ventilation-induced lung injury
V/Q: Ventilation-perfusion
EIT: Electrical impedance tomography
ICU: Intensive care unit
PaO₂: Partial pressure of arterial oxygen
FiO₂: Fraction of inspired oxygen
BMI: Body mass index
IMV: Invasive mechanical ventilation
VV-ECMO: Venovenous extracorporeal membrane oxygenation
PEEP: Positive end-expiratory pressure
SpO_2_: Oxygen saturation
ROIs: Regions of interest
GI: Global inhomogeneity
CoV: Center of ventilation
CRRT: Continuous renal replacement therapy
ANOVA: Analysis of variance
PaCO_2_: Partial pressure of arterial carbon dioxide
CVP: Central venous pressure
ScvO_2_: Central venous oxygen saturation
