# Supplementary material for: Association between ventilation–perfusion matching improvement during initial prone positioning and ICU mortality in patients with moderate to severe ARDS: a prospective two-center study
Source: Ann Intensive Care. 2025 May 21;15:69. doi: 10.1186/s13613-025-01489-1 (PMC12092903; doi:10.1186/s13613-025-01489-1)
Supplement: Supplementary file 1 — Supplementary Material 1: Additional file 1: 1. Online Table 1.1 Table S1. Comparison of ventilatory variables, arterial blood gas, hemodynamic status, and electrical impedance tomography between responder and non-responder groups during the first PP session. 1.2 Table S2 Changes in shunt, dead space, and V/Q matching 4 hours within the first PP in responders and non-responders. 1.3 Table S3 Comparison of echocardiographic variables between responders and non-responders in the supine position before and after PP. 2. Online Figures. 2.1 Figure S1 Flow chart. 2.2 Figure S2 Comparison of PEEP (A), tidal volume (B), driving pressure (C), and respiratory system compliance (D) between responders and non-responders during the first PP session. 2.3 Figure S3 Comparison of PaO2 (A), PaCO2 (B), PaO2/FiO2 (C), and ventilatory ratio (D) between responders and non-responders during the first PP session. 2.4 Figure S4 Comparison of CVP (A) and ScvO2 (B) between responders and non-responders during the first PP session. 2.5 Figure S5 Changes in ventilation (A) and perfusion (B) distribution across four ventral-to-dorsal horizontal regions during the first PP session. [file 13613_2025_1489_MOESM1_ESM.docx]

**ADDITIONAL FILE 1: SUPPLEMENTARY MATERIAL**

**Association between ventilation–perfusion matching improvement during prone positioning and ICU mortality in patients with moderate to severe ARDS: A prospective two-center study**

Rui Wang, MD^1^; Wancong Wang MD^2^; Xiao Tang, MD^1^; Zhenyuan Qi MD^2^; Ting Li MD^1^; Yalan Liu , MD^1^; Hongju Li , MD^2^; Jican Yan, MD^2^; Hua Yang, MD^1^; Wenrui Lyu, MD^1^; Zhaohong Li, MD^1^; Bing Sun, MD^1^; Guifen Gan, MD^2^

**Author Affiliations:**

1. Department of Respiratory and Critical Care Medicine, Beijing Institute of Respiratory Medicine and Beijing Chao-Yang Hospital, Capital Medical University, No. 8 Gongren Tiyuchang Nanlu, Chaoyang District, Beijing, China.
2. Department of Critical Care Medicine, Affiliated Hospital of Qinghai University, Xining, China.

**Corresponding author:**

Guifen Gan MD

Department of Critical Care Medicine, Affiliated Hospital of Qinghai University, No. 29 Tongren Road, Chengdong District, Xining, Qinghai, China.

Email: xzhd1991@163.com

Rui Wang MD

Department of Respiratory and Critical Care Medicine, Beijing Institute of Respiratory Medicine and Beijing Chao-Yang Hospital, Capital Medical University, No. 8 Gongren Tiyuchang Nanlu, Chaoyang District, Beijing, China.

Email: wangrui1985816@gmail.com

**1. Online Tables Supplement**

1.1 Table S1 Comparison of ventilatory variables, arterial blood gas, hemodynamic status, and electrical impedance tomography between responder and non-responder groups during the first PP session

| **Variables** | **Group** | **Supine before PP**  **(n=46/31)** | **During PP**  **(n=46/31)** | **End PP**  **(n=46/31)** | **Supine after PP**  **(n=46/31)** | ***p^a^*** |
| --- | --- | --- | --- | --- | --- | --- |
| **Ventilatory variables** |  |  |  |  |  |  |
| PEEP, mmH_2_O | Responders | 10.8 ± 2.0 | 10.7 ± 1.9 | 10.7 ± 1.9 | 10.7 ± 1.8 | 0.676 |
|  | Non-responders | 10.3 ± 1.8 | 10.2 ± 2.4 | 10.1 ± 2.2 | 10.4 ± 1.8 | 0.439 |
|  | *p^c^* | 0.338 | 0.385 | 0.316 | 0.533 | 0.362*^b^* |
| Tidal volume, ml/PBM | Responders | 6.5 ± 0.6 | 6.4 ± 0.7 | 6.4 ± 0.7 | 6.4 ± 0.7 | 0.485 |
|  | Non-responders | 6.5 ± 1.0 | 6.5 ± 1.0 | 6.5 ± 1.0 | 6.4 ± 1.0 | 0.704 |
|  | *p^c^* | 0.965 | 0.728 | 0.859 | 0.951 | 0.860*^b^* |
| Driving pressure, cmH_2_O | Responders | 12.8 ± 3.6 | 12.1 ± 3.4 | 11.2 ± 3.0 | 11.7 ± 3.0 | < 0.001 |
|  | Non-responders | 12.1 ± 3.3 | 12.0 ± 3.7 | 11.8 ± 3.5 | 11.8 ± 3.8 | 0.707 |
|  | *p^c^* | 0.457 | 0.953 | 0.518 | 0.474 | 0.970*^b^* |
| Compliance, ml/cmH_2_O | Responders | 34.4 ± 9.4 | 35.7 ± 8.9 | 38.3 ± 8.8 | 36.7 ± 8.7 | < 0.001 |
|  | Non-responders | 36.8 ± 7.2 | 37.1 ± 7.7 | 37.5 ± 8.2 | 37.8 ± 8.5 | 0.728 |
|  | *p^c^* | 0.273 | 0.265 | 0.729 | 0.626 | 0.632*^b^* |
| **Arterial blood gas** |  |  |  |  |  |  |
| PaO_2_, mmHg | Responders | 67.6 ± 11.5 | 77.2 ± 10.3 | 85.1 ± 11.8 | 80.1 ± 8.7 | < 0.001 |
|  | Non-responders | 71.3 ± 11.8 | 79.1 ± 9.0 | 81.2 ± 8.2 | 77.9 ± 6.7 | < 0.001 |
|  | *p^c^* | 0.237 | 0.446 | 0.165 | 0.143 | 0.953*^b^* |
| PaCO_2_, mmHg | Responders | 42.3 ± 10.6 | 40.6 ± 11.0 | 39.5 ± 11.1 | 40.3 ± 11.2 | < 0.001 |
|  | Non-responders | 41.0 ± 8.2 | 40.1 ± 8.6 | 39.9 ± 8.2 | 40.9 ± 8.2 | < 0.001 |
|  | *p^c^* | 0.593 | 0.870 | 0.883 | 0.810 | 0.941*^b^* |
| PaO_2_:FiO_2_ ratio, mmHg | Responders | 111.8 ± 26.4 | 173.1 ± 25.4 | 193.9 ± 35.5 | 178.0 ± 27.8 | < 0.001 |
|  | Non-responders | 115.3 ± 22.9 | 145.4 ± 20.6 | 155.4 ± 25.2 | 140.2 ± 21.1 | < 0.001 |
|  | *p^c^* | 0.595 | < 0.001 | < 0.001 | < 0.001 | < 0.001*^b^* |
| Ventilatory ratio | Responders | 1.60 (1.32 - 1.91) | 1.49 (1.27 - 1.96) | 1.42 (1.18 - 1.84) | 1.43 (1.16 - 1.80) | 0.008 |
|  | Non-responders | 1.55 (1.33 - 1.92) | 1.44 (1.15 - 1.86) | 1.36 (1.15 - 1.79) | 1.54 (1.38 - 1.97) | < 0.001 |
|  | *p^c^* | 0.785 | 0.322 | 0.802 | 0.137 | 0.847*^b^* |
| **Hemodynamic status** |  |  |  |  |  |  |
| CVP, mmHg | Responders | 9 (7 -13) | 12 (10 - 15) | 13 (10 - 14) | 11 (8 - 12) | 0.004 |
|  | Non-responders | 11 (9 - 15) | 11 (9 - 14) | 13 (10 - 16) | 10 (8 - 12) | 0.093 |
|  | *p^c^* | 0.195 | 0.312 | 0.272 | 0.544 | 0.654*^b^* |
| ScvO_2_, % | Responders | 57.3 ± 11.4 | 66.3 ± 10.3 | 73.7 ± 12.5 | 68.3 ± 8.4 | < 0.001 |
|  | Non-responders | 62.7 ± 10.7 | 68.7 ± 8.5 | 68.4 ± 8.6 | 66.0 ± 7.3 | 0.084 |
|  | *p^c^* | 0.068 | 0.335 | 0.070 | 0.267 | 0.984*^b^* |
| **Electrical impedance tomography** |  |  |  |  |  |  |
| GI index ventilation | Responders | 0.59 (0.56 - 0.65) | 0.52 (0.49 - 0.53) | 0.49 (0.47 - 0.52) | 0.52 (0.49 - 0.54) | < 0.001 |
|  | Non-responders | 0.59 (0.55 - 0.62) | 0.53 (0.51 - 0.57) | 0.50 (0.48 - 0.53) | 0.52 (0.50 - 0.55) | < 0.001 |
|  | *p^c^* | 0.416 | 0.002 | 0.213 | 0.485 | 0.113*^b^* |
| Center of ventilation, % | Responders | 44.62 ± 6.21 | 52.75 ± 5.65 | 53.30 ± 9.43 | 48.69 ± 5.79 | < 0.001 |
|  | Non-responders | 43.55 ± 5.09 | 49.00 ± 4.13 | 51.54 ± 6.67 | 46.48 ± 5.99 | < 0.001 |
|  | *p^c^* | 0.478 | 0.006 | 0.423 | 0.152 | 0.031*^b^* |
| Shunt, % | Responders | 22.3 ± 10.3 | 15.4 ± 7.7 | 14.6 ± 8.8 | 17.3 ± 11.8 | 0.003 |
|  | Non-responders | 21.9 ± 8.5 | 21.3 ± 11.5 | 20.9 ± 10.9 | 21.7 ± 10.3 | 0.970 |
|  | *p^c^* | 0.900 | 0.019 | 0.015 | 0.134 | 0.023*^b^* |
| Dead space, % | Responders | 24.5 ± 10.9 | 15.1 ± 7.8 | 12.6 ± 7.1 | 18.4 ± 12.1 | < 0.001 |
|  | Non-responders | 20.9 ± 10.1 | 16.1 ± 5.7 | 15.0 ± 10.7 | 20.0 ± 12.6 | 0.024 |
|  | *p^c^* | 0.184 | 0.607 | 0.170 | 0.623 | 0.853*^b^* |
| V/Q matching, % | Responders | 56.5 ± 7.8 | 73.3 ± 6.4 | 76.5 ± 7.9 | 64.8 ± 11.5 | < 0.001 |
|  | Non-responders | 62.5 ± 8.7 | 68.9 ± 7.8 | 64.3 ± 10.9 | 58.3 ± 12.9 | < 0.001 |
|  | *p^c^* | 0.008 | 0.017 | < 0.001 | 0.040 | 0.037*^b^* |

PP prone positioning, PEEP positive end-expiratory pressure, PBM predicted body weight, PaO_2_ partial pressure of arterial oxygen, PaCO_2_ partial pressure of arterial carbon dioxide, FiO_2_ the fraction of inspired oxygen, CVP central venous pressure, ScvO_2_ central venous oxygen saturation, GI, global Inhomogeneity, V/Q ventilation/perfusion

*p^a^* for overall comparisons of differences in each group over time.

*p^b^* for overall comparisons of differences between groups over time.

*p^c^* for comparisons of differences between groups at each time point.

1.2 Table S2 Changes in shunt, dead space, and V/Q matching 4 hours within the first PP in responders and non-responders

| **Variables** | **Responders (n = 46)** | **Non-responders (n = 31)** | ***P*** |
| --- | --- | --- | --- |
| Δ Shunt, % | -6.8 ± 10.3 | -0.6 ± 9.9 | 0.021 |
| Δ Dead space, % | -9.4 ± 11.2 | -5.2 ± 9.2 | 0.125 |
| Δ V/Q matching, % | 16.1 ± 5.2 | 6.4 ± 4.5 | < 0.001 |

PP prone positioning, V/Q ventilation/perfusion

1.3 Table S3 Comparison of echocardiographic variables between responders and non-responders in the supine position before and after PP.

| **Variables** | **Responders (n = 46)** | **Non-responders (n = 31)** | ***P*** |
| --- | --- | --- | --- |
| **Supine position before PP** |  |  |  |
| Right ventricle |  |  |  |
| Right ventricular : left ventricular end-diastolic area | 0.56 (0.51 - 0.70) | 0.60 (0.49 - 0.69) | 0.908 |
| Right ventricular fractional area change, % | 40.2 (32.5 - 46.8) | 41.2 (31.7 - 50.4) | 0.731 |
| Tricuspid annular plane systolic excursion, mm | 21 (19 - 23) | 20 (18 - 24) | 0.713 |
| Maximum tricuspid regurgitation velocity, m/s | 259 (204 - 265) | 253 (216 - 274) | 0.802 |
| Left ventricle |  |  |  |
| Ejection fraction, % | 64 (53 - 69) | 65 (57 - 71) | 0.620 |
| Stroke volume, ml | 70 (58 - 88) | 75 (55 - 85) | 0.846 |
| Cardiac output, L/min | 5.3 (4.0 - 7.5) | 5.5 (3.9 - 7.5) | 0.863 |
| Inferior vena cava diameter, cm | 1.9 (1.4 - 2.3) | 1.8 (1.5 - 2.2) | 0.801 |
| Collapsibility inferior vena cava, % | 30.8 (4.9 - 52.0) | 22.9 (7.5 - 41.0) | 0.725 |
| **Supine position after PP** |  |  |  |
| Right ventricle |  |  |  |
| Right ventricular : left ventricular end-diastolic area | 0.50 (0.45 - 0.67) | 0.59 (0.47 - 0.66) | 0.408 |
| Right ventricular fractional area change, % | 42.7 (35.9 - 48.3) | 39.9 (33.4 - 46.7) | 0.295 |
| Tricuspid annular plane systolic excursion, mm | 21 (19 - 25) | 20 (18 - 23) | 0.302 |
| Maximum tricuspid regurgitation velocity, m/s | 257 (222 - 261) | 249 (215 - 259) | 0.813 |
| Left ventricle |  |  |  |
| Ejection fraction, % | 65 (56 - 70) | 62 (53 - 72) | 0.914 |
| Stroke volume, ml | 72 (55 - 86) | 69 (59 - 87) | 0.790 |
| Cardiac output, L/min | 5.9 (4.4 - 7.4) | 5.2 (4.0 - 7.1) | 0.500 |
| Inferior vena cava diameter, cm | 1.9 (1.5 - 2.3) | 1.8 (1.5 - 2.2) | 0.326 |
| Collapsibility inferior vena cava, % | 20.3 (2.7 - 51.8) | 16.2 (8.3 - 42.3) | 0.886 |

PP prone positioning

**2. Online Figures Supplement**

2.1 Figure S1 Flow chart


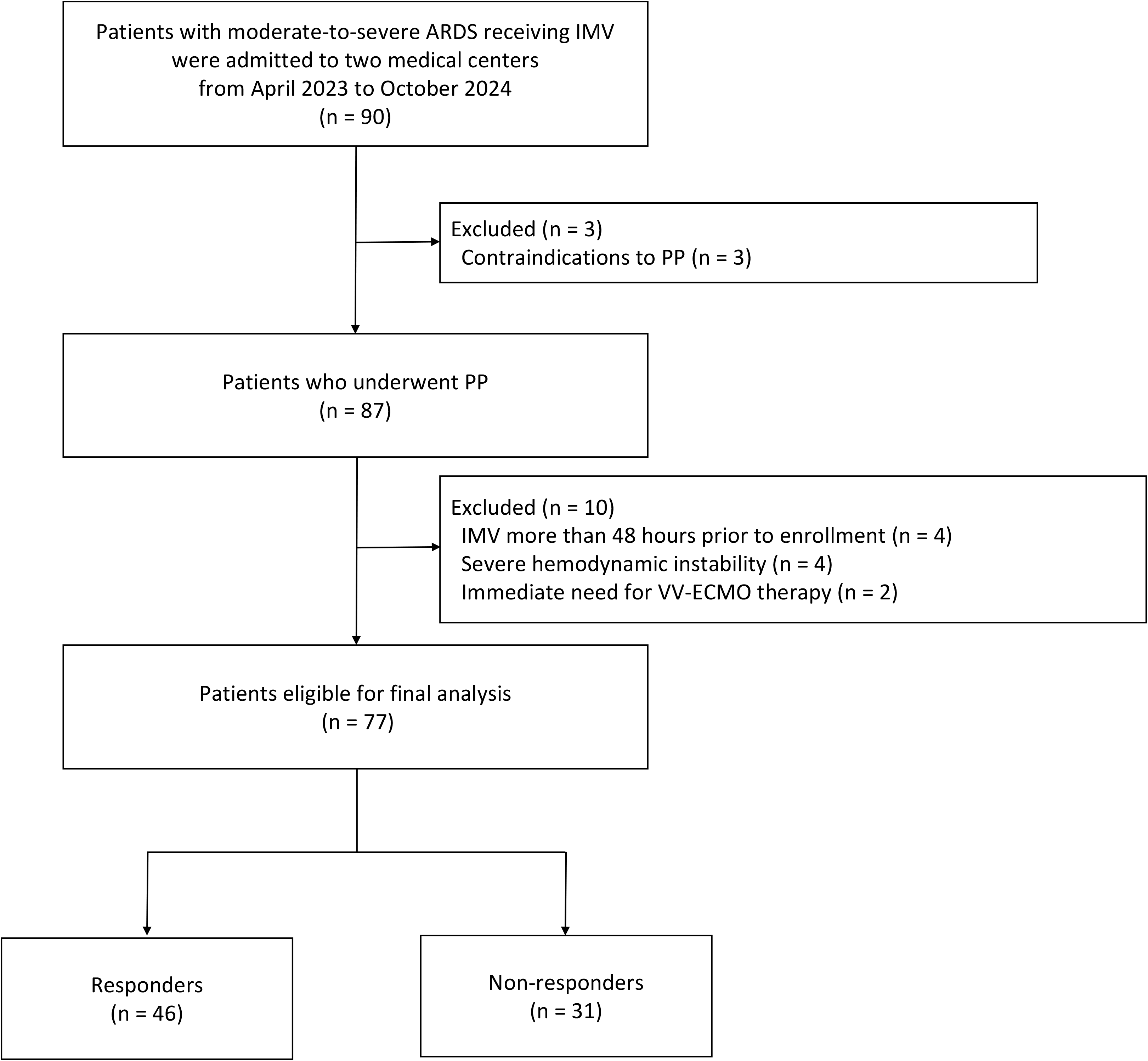


ARDS acute respiratory distress syndrome, IMV invasive mechanical ventilation, PP prone positioning, VV-ECMO venovenous extracorporeal membrane oxygenation

2.2 Figure S2 Comparison of PEEP **(A)**, tidal volume **(B)**, driving pressure **(C)**, and respiratory system compliance **(D)** between responders and non-responders during the first PP session


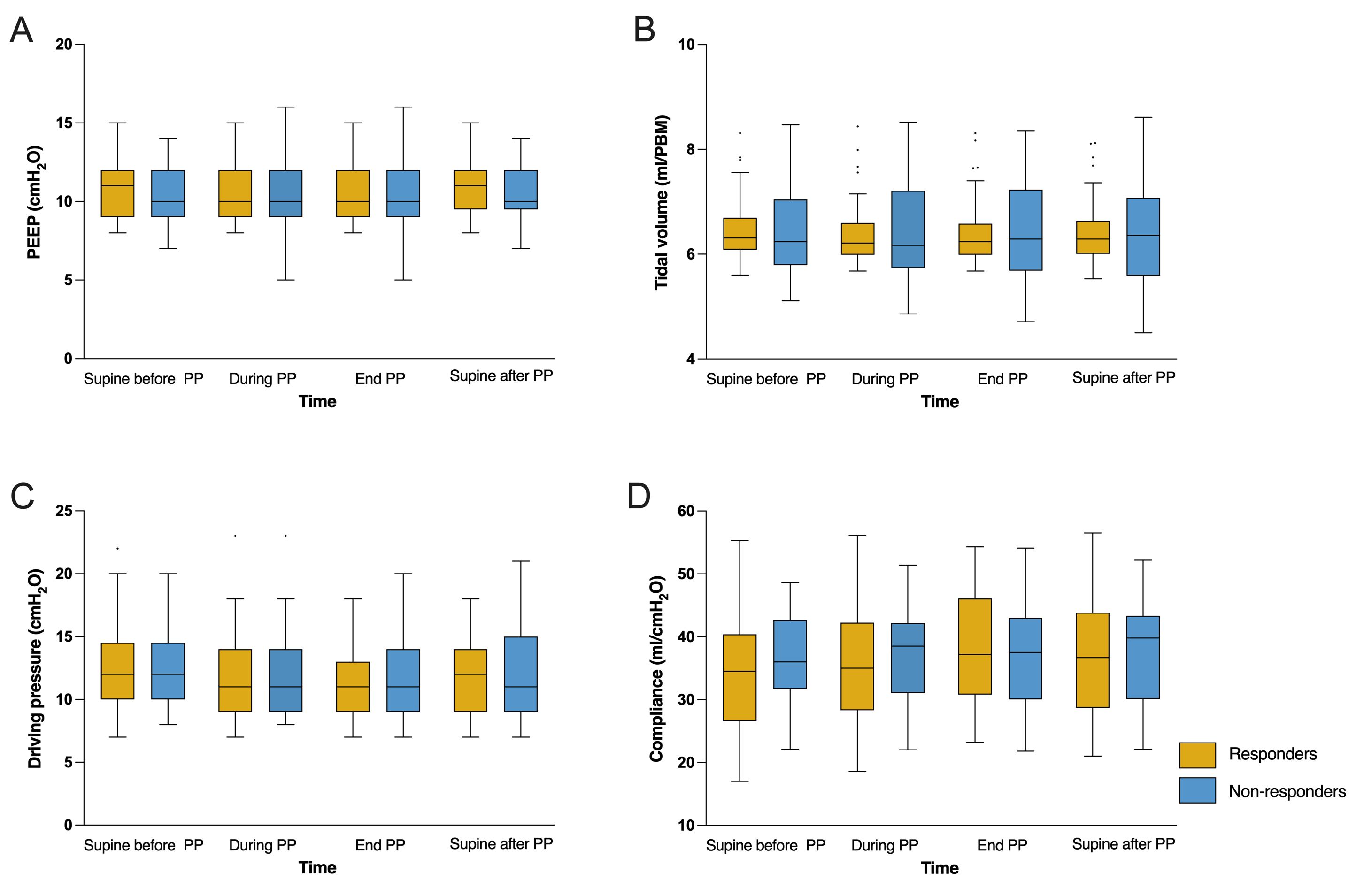


PP prone positioning, PEEP positive end-expiratory pressure, PBM predicted body weight

2.3 Figure S3 Comparison of PaO_2_ **(A)**, PaCO_2_ **(B)**, PaO_2_/FiO_2_ **(C)**, and ventilatory ratio **(D)** between responders and non-responders during the first PP session.


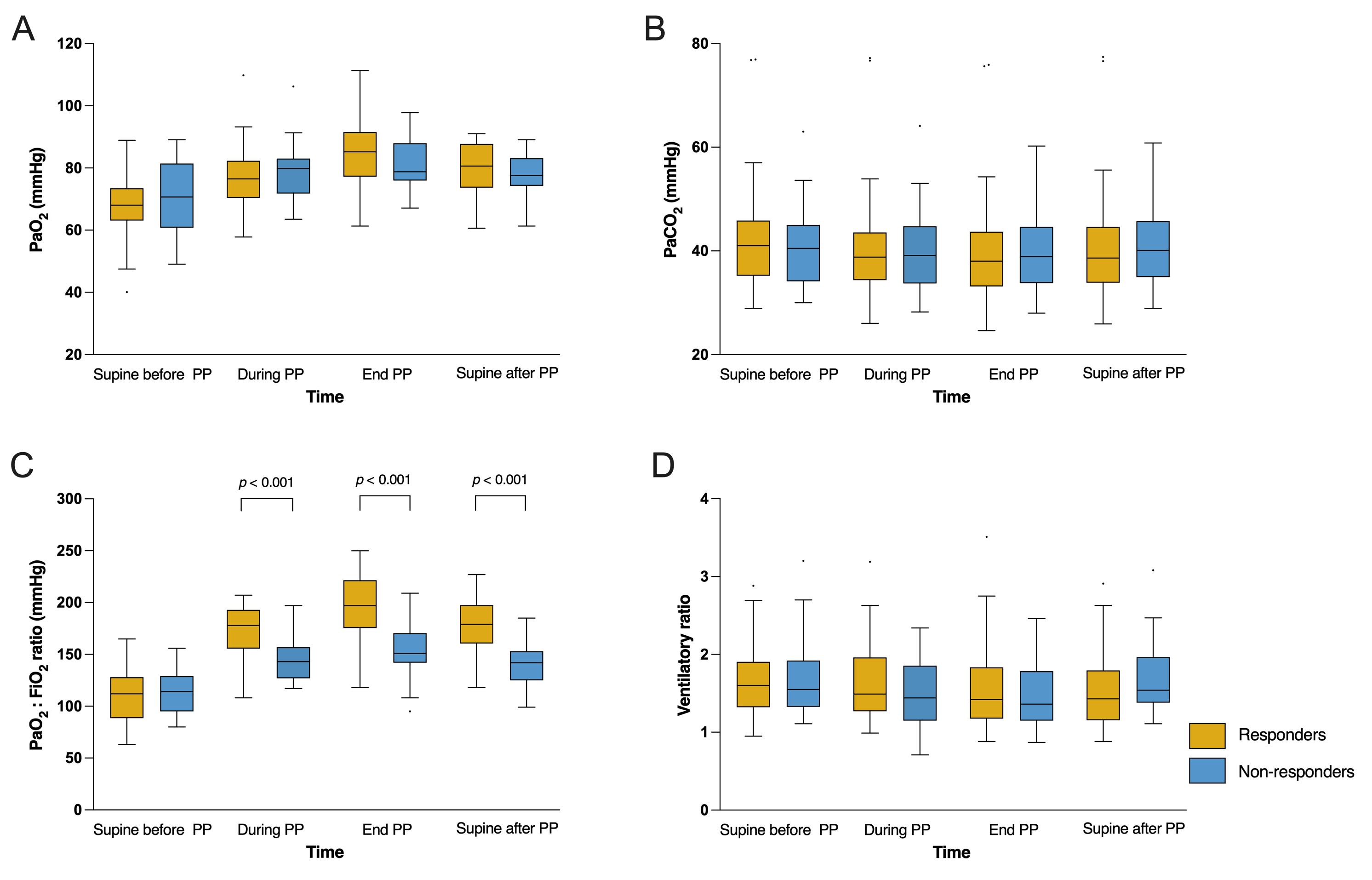


PaO_2_ partial pressure of arterial oxygen, PaCO_2_ partial pressure of arterial carbon dioxide, FiO_2_ the fraction of inspired oxygen, PP prone positioning

2.4 Figure S4 Comparison of CVP **(A)** and ScvO_2_ **(B)** between responders and non-responders during the first PP session.


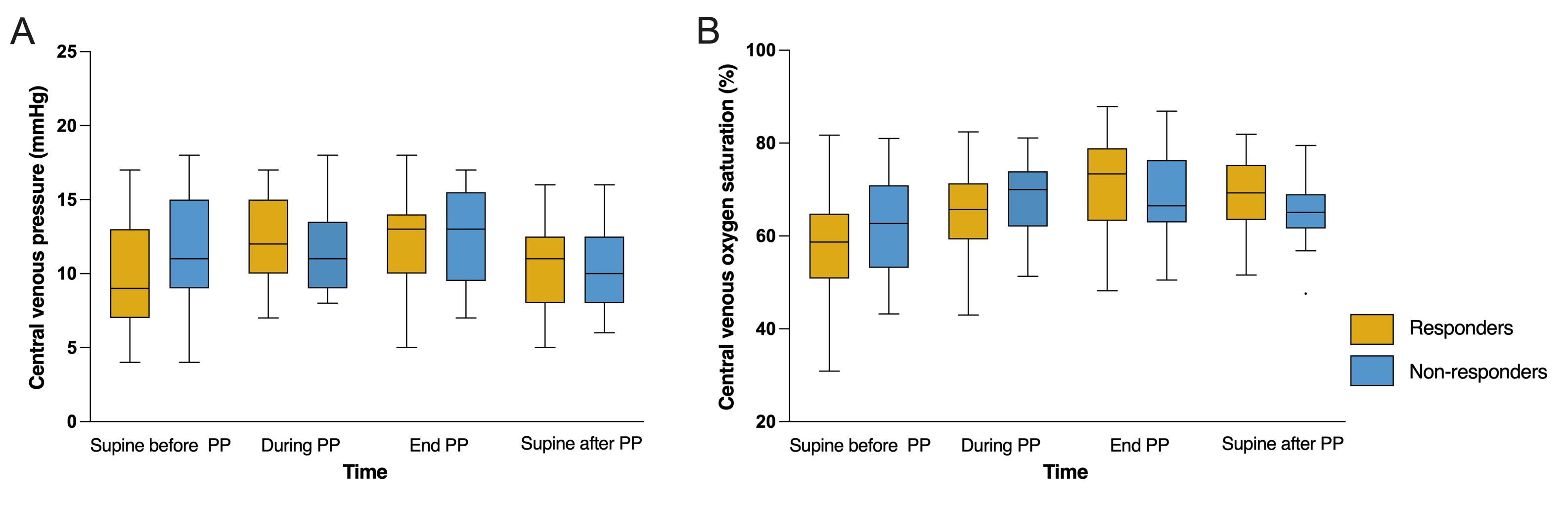


CVP central venous pressure, ScvO_2_ central venous oxygen saturation

2.5 Figure S5 Changes in ventilation **(A)** and perfusion **(B)** distribution across four ventral-to-dorsal horizontal regions during the first PP session.


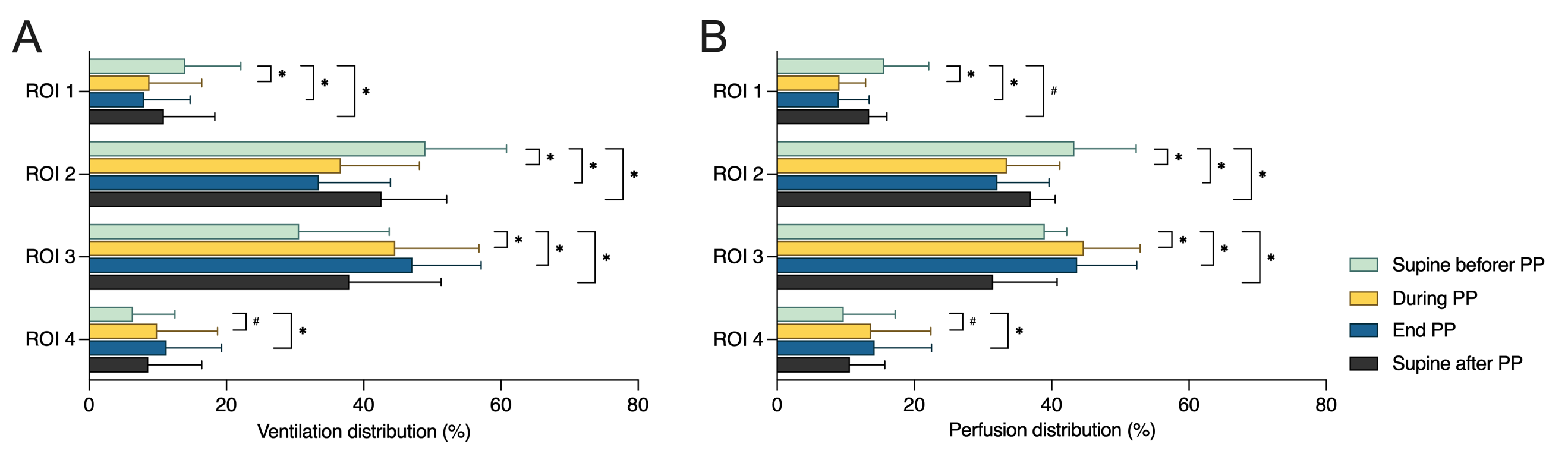


PP prone positioning, ROI regions of interest

^#^ *P <*0.05, * *P <*0.01.
